# Phenotypic and Genotypic Characterization of DEPDC5-Related Familial Focal Epilepsy: Case Series and Literature Review

**DOI:** 10.3389/fneur.2021.641019

**Published:** 2021-06-22

**Authors:** Xuan Zhang, Zhaoyang Huang, Jianghong Liu, Mingyu Li, Xiaoling Zhao, Jing Ye, Yuping Wang

**Affiliations:** ^1^Department of Neurology, Xuanwu Hospital, Capital Medical University, Beijing, China; ^2^Beijing Key Laboratory of Neuromodulation, Beijing, China; ^3^Institute of Sleep and Consciousness Disorders, Beijing Institute of Brain Disorders, Collaborative Innovation Center for Brain Disorders, Capital Medical University, Beijing, China

**Keywords:** penetrance, refractory epilepsy, genotype, phenotype, family focal epilepsy, DEPDC5

## Abstract

Mutations in the disheveled, Egl-10 and domain-containing protein 5 (DEPDC5) recently have been identified as a common cause of focal epilepsy syndromes. The association between phenotype and genotype of DEPDC5 mutation has not been adequately characterized. We studied four families with familial focal epilepsy carrying DEPDC5 mutations. Four novel DEPDC5 mutations were identified by next-generation sequencing, including two missense mutations (c.1729 >A and c.3260G>A), one splicing mutation (c.280-1G>A), and one frameshift mutation (c.515_516delinsT). We found that patients carrying different DEPDC5 mutation have different clinical manifestations. Incomplete penetrance is a prominent feature of DEPDC5-related epilepsy, with the rate of penetrance ranging from 25 to 100%. About 21.4% of patients with DEPDC5-related familial focal epilepsy are refractory to treatments. We further reviewed the correlation of genotype and phenotype in all previous literature regarding DEPDC5-related epilepsy. Our study suggested that the type of DEPDC5 mutation might provide important information for the prognosis evaluation.

## Introduction

Focal epilepsy is the most common type of epilepsy, accounting for 60–70% of all epilepsy cases. However, the etiology of more than half of the focal epilepsies remains unclear. Recent studies found that genetic factor plays a major role in the pathogenesis of focal structural epilepsies (1). Mutations in the disheveled, Egl-10 and domain-containing protein 5 (DEPDC5) have emerged as an important cause of focal epilepsy. DEPDC5 mutations have been identified in 5% of sporadic epilepsy and 13% of familial epilepsy (2, 3). DEPDC5-related epilepsy comprises a range of epilepsy syndromes, including familial focal epilepsy (FFE) with variable foci (FFEVF), autosomal dominant sleep-related hypermotor epilepsy (ADSHE), familial mesial and lateral temporal lobe epilepsy (TLE), benign epilepsy with centrotemporal spikes (BECTS), and infantile spasms (4, 5).

GTPase-activating protein (GAP) activity toward RAGs (GATOR) complex and tuberous sclerosis complex (TSC) act as critical regulators of the mechanistic target of rapamycin (mTOR). GATOR consists of two subcomplexes, GATOR1 and GATOR2. DEPDC5 is a part of GATOR1 and, together with the proteins NPRL2 and NPRL3, exerts an inhibitive influence on the mTOR signaling pathway (6, 7). GATOR2, on the contrary, plays a positive role in the mTORC1 signaling pathway by selectively inhibiting DEPDC5. mTOR, consisting of mTORC1 and mTORC2, is an essential regulator in cell proliferation and brain growth. Deregulation of the mTOR pathway has always been linked to malformation of cortical development (MCD), such as focal cortical dysplasia (FCD), and hemimegalencephaly. GATOR1 inhibits mTORC1 signaling when local amino acid (AA) levels are limited, whereas GATOR2 blocks GATOR1 in the presence of increasing local AA, resulting in mTORC1 hyperactivity (8). DEPDC5 dysfunction results in activation of the mTORC1 signaling pathway, leading to abnormal neuronal morphology, aberrant cortical laminar structure, cortical neuronal hyperexcitability, and eventually clinical seizures.

Clinical heterogeneity was noticed in patients carrying different mutations of DEPDC5. Several studies have reported DEPDC5-related epilepsy. However, most of them only emphasize the molecular level. Clarifying the genotype–phenotype relationship of DEPDC5 mutations is important for customizing treatment strategies and evaluating the prognosis. In this study, we described the clinical characteristics of four families with different novel mutations in DEPDC5. Furthermore, we reviewed the advanced publications and summarized the associations between phenotype and genotype in DEPDC5-related epilepsy.

## Methods

### Research Subjects and Clinical Evaluation

All procedures of this study were approved by the Institutional Review Board of Xuanwu Hospital. Written informed consents were obtained from the individuals and minors' legal guardian for the publication of any potentially identifiable images or data included in this article.

Our study involved four Chinese families clinically diagnosed with FFE. All participates were admitted to Xuanwu Hospital of Capital Medical University. Our inclusion criteria included (1) confirmed diagnosis of focal epilepsy based on the criteria of the International League Against Epilepsy (ILAE); (2) family history of epilepsy; and (3) pathogenic/likely pathogenic DEPDC5 mutation in the affected family members. All the probands, as well as their affected and non-affected family members, received comprehensive clinical evaluations by experienced neurologists in our hospital. Brain magnetic resonance imaging (MRI) and electroencephalogram (EEG) were performed on these participants. Auxiliary examinations, including liver and kidney function, blood and urine organic acids, and electrolytes, were performed.

### Genetic Study

To extract DNA of some affected and unaffected members from four families, 2 ml of venous blood of the participants was collected and placed in the anticoagulant tube containing ethylenediamine tetraacetic acid (EDTA). Genomic DNA of blood samples was extracted by Qiagen flexigene DNA Kit (product No. 51206, Qiagen Company, Germany) and stored at −20°C. DNA extraction procedures were carried out in strict accordance with the instructions of the kit. With reference to OMIM and HGMD, we selected 1,553 genes related to various types of epilepsy and related paroxysmal diseases. Using the online design tool of Agilent (Agilent company), we designed a targeted capture probe for the exon and flanking sequence (±10 bp) of the target gene and customized a special target gene capture kit, which was shared by the library 141,316 capture probes with a total size of 6.758 Mbp. We used Illumina's NextSeq 500 amplified products to conduct single-ended sequencing and then compared the sequencing read to the human reference genome (hg19 version), using BWA (v0.7.15). RefSeq, Ensembl, UCSC, and other databases were used for gene annotation. Note the frequency of variation in the population by searching 1000G, dbSNP, ESP6500, EXAC, genomeAD, HGMDpro, etc. Protein damage analysis was performed with Polyphen, M-CAP, SIFT, and the like, and mutations were graded by the American College of Medical Genetics (ACMG). Different samples were analyzed separately, including clinical symptom matching and genetic pattern matching. The Sanger was carried out for the mutation that needs to be verified in samples.

### Literature Search

A systematic literature search in PubMed was performed. MeSH and title/abstract were used for all eligible studies that mainly focus on the DEPDC5 mutation in FFE. The research strategy was as follows: “DEPDC5” AND (“familial focal epilepsy” or “familial focal epilepsy with variable foci” or “autosomal dominant sleep-related hypermotor epilepsy” or “familial mesial and lateral temporal lobe epilepsy” or “familial epilepsy”). Eligible studies must meet the following criteria: (1) at least two family members were diagnosed with focal epilepsy; and (2) a pathogenic/likely pathogenic DEPDC5 mutation was identified. Sporadic cases of DEPDC5-related epilepsy were excluded. Data from all eligible studies were analyzed and discussed by two reviewers.

## Results

### Clinical Manifestations

**Figure 1:**
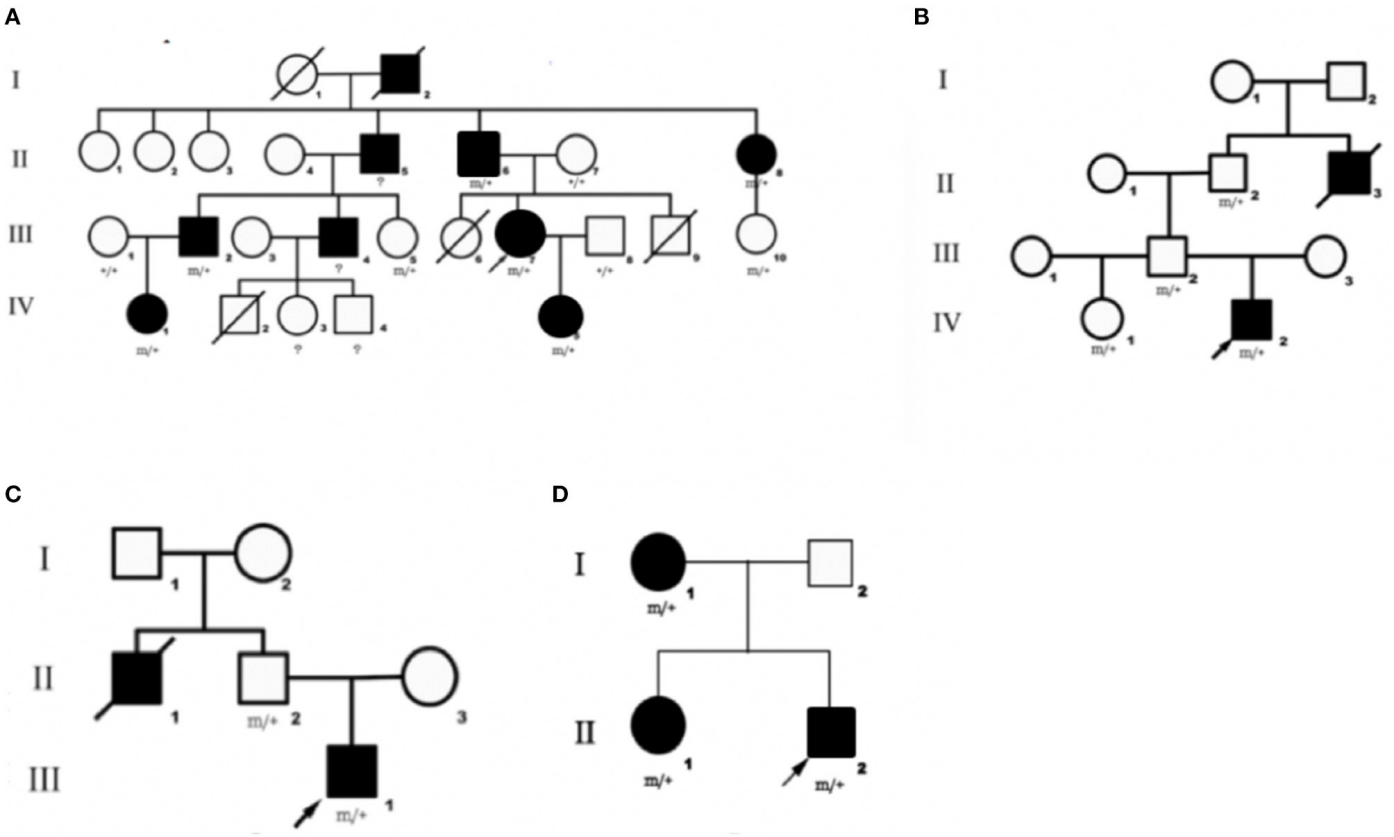
Pedigrees of four families diagnosed with familial focal epilepsy. **(A–D)** Families 1–4, respectively. Black squares/circles indicate male/female members with epilepsy in the family. Deaths are indicated by slashes. Probands are indicated by oblique arrows. Individuals carrying the DEPDC5 mutation are indicated by (m/+). Individuals with negative mutation are indicated by (+/+), and individuals without blood samples are indicated by (?).

We identified DEPDC5 mutations in 10 patients and six asymptomatic carriers from four families. The pedigrees of four families are shown in Figures 1A–D. Participant's clinical features are listed in Table 1.

**Table 1:** Clinical characteristics of patients carrying DEPDC5 mutations from four families with familial focal epilepsy.

**No**	**Age (onset)**	**Gender**	**Type of epilepsy**	**Intellectual disability**	**AED (used)**	**Effective AED**	**Drug resistant**	**VEEG**	**MRI**	**DEPDC5 mutation**
Family1										
II-6	20 years	M	FLE	N	OXC, PB, PHT		Yes	ND	N	c.280-1G>A
III-7	3 months	F	TLE	N	CBZ, LTG, VPA, CZP		Yes	Sharp wave in the right anterior and middle temporal	N	c.280-1G>A
IV-5	3 months	F	OLE	Y	OXC, VPA, LTG, PB, PHT		Yes	Occasional low-to-medium amplitude spikes on both sides of the frontal and central	N	c.280-1G>A
Family2										
IV-2	24 years	M	OLE	N	OXC	OXC	No	Left occipital sharp wave	N	c.1729G>A
Family3										
III-1	2 years	M	TLE	N	LEV, OXC	LEV, OXC	No	Sharp waves in the left frontal pole, anterior temporal, and sphenoid electrodes	N	c.515_516delinsT
Family4										
I-1	45 years	F	FLE	N			No	ND	ND	c.3260G>A
II-1	5 years	F	FE	N	CBZ	CBZ	No	ND	ND	c.3260G>A
II-2	9 years	M	FLE	N	VPA, LTG, PB, PHT, CBZ		Yes	Mild abnormalities	N	c.3260G>A

*F, female; M, male; N, normal; OXC, oxcarbazepine; PB, phenobarbital; PHT, phenytoin; VPA, valproate; LEV, levetiracetam; CBZ, carbamazepine; LTG, lamotrigine; CZP, clonazepam; FLE, frontal lobe epilepsy; TLE, temporal lobe epilepsy; OLE, occipital lobe epilepsy; PLE, parietal lobe epilepsy; FE, focal epilepsy; ND, not done; AED, anti-epileptic drug; VEEG, video-electroencephalogram*.

#### Family 1

This family (9) was clinically diagnosed with FFEVF. The proband (III-7) suffered from TLE characterized by loss of consciousness, preceded by déjà vu occasionally. The proband's daughter (IV-5) experienced paroxysmal attacks of brief clouding of consciousness and sometimes tonic–clonic seizures. She also had profound developmental delay. The proband's father (II-6) was diagnosed with frontal lobe epilepsy. The proband's grandfather (I-2), uncle (II-5), aunt (II-8), two cousins (III-2 and III-4), and nephew (IV-1) have a history of epilepsy, and they have been seizure-free since middle age after treatment.

#### Family 2

Mutation of DEPDC5 was identified in four family members, whereas only two of them have symptoms. The proband (IV-2), a 24-year-old young male, presented with paroxysmal unconsciousness and limb stiffness. Oxcarbazepine (OXC) was effective, but this patient still has seizure attacks after exhaustion. His uncle died of epilepsy at an early age, presumably from sudden unexpected death in epilepsy (SUDEP).

#### Family 3

We identified DEPDC5 mutations in two patients and two unaffected carriers from this family. The proband (III-1) experienced paroxysmal unconsciousness and limb stiffness preceded by an aura of fear. He had febrile seizures at 1 year of age. The proband's uncle (II-1) has a history of epilepsy and died at 36 years old. And probable SUDEP was suspected in this patient.

#### Family 4

There were three patients in this family. The proband (II-2) experienced seizures characterized by shouting and limb stiffness without awareness. He is refractory to multiple anti-epileptic drugs (AEDs). The proband's elder sister (II-1) suffered from tonic–clonic seizures at 5 years old. The proband's mother (I-1) presented tonic seizures at night once a year.

### Novel Mutations in DEPDC5 Gene

**Figure 2:**
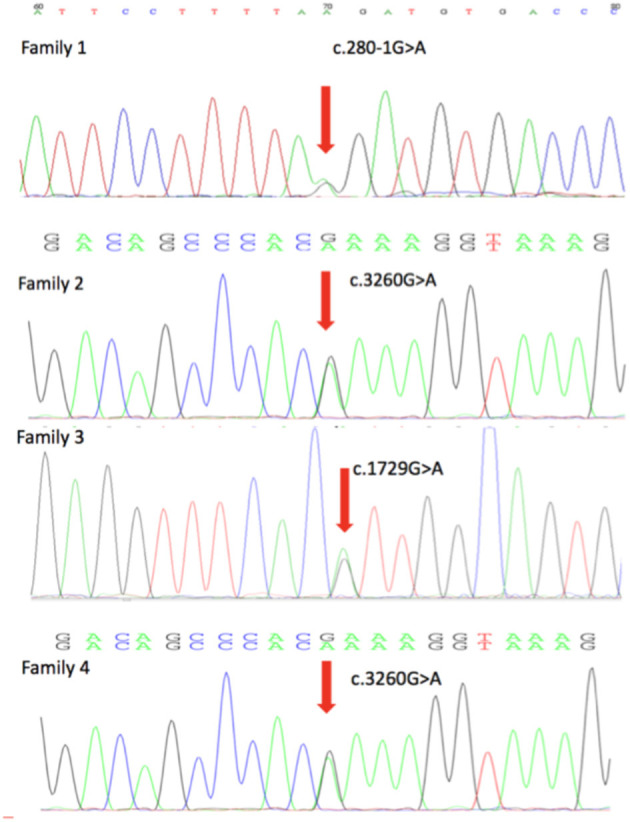
DEPDC5 mutations in the four focal epilepsy families. DNA sequencing chromatograms showed the mutations in DEPDC5 identified in these four families. Red arrows point to the positions of DEPDC5 mutations.

Four novel mutations in the DEPDC5, including c.280-1G>A, c.1729G>A, c.515_516delinsT, and c.3260G>A, were identified by second-generation sequencing. All the mutations were verified by Sanger sequencing. The variant c.280-1G>A is a splicing mutation found in family 1, which affects the structure of mRNA and results in loss of protein function. The allele frequency of the variant c.280-1G>A in East Asian population is 0. According to the criteria of the ACMG, the variant c.280-1G>A was classified as likely pathogenic. The variant c.1729G>A and c.3260G>A were identified in family 2 and family 4, respectively. The variant c.3260G>A was classified as variant of unknown significance (VoUS). However, when we used Mendelian Clinically Applicable Pathogenicity (M-CAP) to assess its pathogenicity, it was classified as possibly pathogenic. The allele frequency of this variant in East Asian population is 0. We used the ACMG and M-CAP to assess the pathogenicity of c.1729G>A, which is classified as VoUS. The allele frequency of the c.1729G>A in East Asian population is also 0. The variant c.515_516delinsT was found in family 3, which is a frameshift mutation. The variant c.515_516delinsT was not found in genomAD and HGMDpro. And it was classified as possibly pathogenic. Sequencing chromatogram of the mutation in DEPDC5 of these four families is listed in Figure 2.

### Results on Literature Search

A total of 48 papers were included after the initial search with the keywords. However, 12 studies with 67 families were included in this review. Table 1 shows the summary of previously reported phenotypes about DEPDC5-related FFE. These studies (Table 2) demonstrated that 52% (35/67) patients carried nonsense mutations of DEPDC5, 20.8% (14/67) patients carried missense mutations of DEPDC5, and 14.2% (10/67) patients carried splicing mutations of DEPDC5. Non-sense mutation and missense mutation are the most common variants.

**Table 2:** Summary of previous reported phenotype about DEPDC5-related familial focal epilepsy.

**DEPDC5 mutation**	**Mutation type**	**Type of epilepsy**	**Drug resistant**	**Other manifestation**	**MRI**	**Penetrance (%)**	**References**
c.21C>G p.Tyr7*	Non-sense	FFEVF	NA	AS, ID	NA	82	(10)
c.1663C>T p.Arg555*	Non-sense	FFEVF	NA	PD	NA	56	
c.488-490delTGT p.Phe164del	Deletion	FFEVF	NA	No	NA	67	
	Deletion	FFEVF	NA	No	NA	53	
	Deletion	FFEVF	NA	PD	NA	81	
c.4107G>A p.Trp1369*	Non-sense	FFEVF	NA	No	NA	64	
c.4606C>T p.Gln1536*	Non-sense	FFEVF	NA	PD, ID	NA	50	
c.4397G>A p.Trp1466*	Non-sense	FFE	NA	No	NA	83	
c.193+1G>A	Splicing	FFE	NA	No	NA	80	
c.279+1G>A	Splicing	FFE	NA	No	NA	100	
c.1459C>T p.Arg487*	Non-sense	FFE	NA	No	NA	66	
c.2527C>T p.Arg843*	Non-sense	FFE	NA	No	NA	100	
c.4397G>A p.Trp1466*	Non-sense	FFE	NA	No	NA	66	
c.3802C>T p.Arg1268*	Non-sense	FFE	NA	No	NA	50	
c.3311C>T p.Ser1104Leu	Missense	FFE	NA	No	NA	100	
c.3217A>C p.Ser1073Arg	Missense	FFE	NA	No	NA	100	
c.1355C>T p.Ala452Val	Missense	FFE	NA	No	NA	100	
c.1122delA p.Leu374Phefs*30	Frameshift	FFEVF	NA	NA	N	50	(11)
c.715C>T p.Arg239*	Non-sense	FFEVF	NA	NA	N	58	
c.982C>T p.Arg328*	Non-sense	FTLE	NA	NA	N	100	
c.1114C>T p.Gln372*	Non-sense	FFEVF	NA	NA	N	100	
c.1454G>A p.Arg485Gln	Missense	FTLE	NA	NA	N	100	
c.4567C>T p.Gln1523*	Non-sense	ADNFLE	NA	NA	N	100	
c.3417delA p.I1139Mfs*24	Frameshift	RE	No	No	NA	50	(12)
c.59-1G>C	Splicing	FFE	No	No	N	66	
c.2593C>T p.R865*	Non-sense	FFE	Yes	DD	NA	75	
c.727C>T p.R243*	Non-sense	FFE	Yes	No	NA		
c.814G>T p.V272L	Missense	RE	No	No	NA	50	
c.268G>A p.V90I	Missense	RE	No	No	NA	50	
c.3457A>G p.S1153G	Missense	RE	No	No	NA	50	
c.2527C>T, p.R843X	Non-sense	FFEVF	NA	No	NA	45	(13)
		FFEVF	NA	No	NA	50	
		FFEVF	NA	No	NA	67	
c.2591C>T; p.T864M	Missense	FFEVF	NA	No	NA		
c.418C>T; p.Gln140*	Non-sense	FFE	Yes	ID, PD	FCD	50	(1)
C.21C>G p.Tyr7*	Non-sense	FFEVF	No	ASD	FCD	88	
c.279+1G>A	Splicing	FFE	No	No	FCD	100	
c.2355-2A>G	Splice	ADNFLE	No	ID, PD	No		(14)
c.1459C>T p.Arg487*	Non-sense	ADNFLE	Yes	ID, PD	No		
c.3259C>T p.Arg1087*	Non-sense	ADNFLE	Yes	ID, PD	No		
c.4107G>A p.Trp1369*	Non-sense	ADNFLE	Yes	ID, PD	No		
Germline:c.715C>T p.Arg239*(Somatic:c.1264C>Tp.Arg422* in brain)	Non-sense	FFEVF	Yes	ID, PD	FCD IIb	58	(15)
c.484-1G>A	Splicing	FFE	Yes	PD	FCD IIa	66	
c.1264C>T p.Arg422*	Non-sense	FFE	Yes	No	FCD I		
c.1759C>T p.Arg587*	Non-sense	FFE	Yes	PD	FCD IIa		
c.918C>G p.Tyr306*	Non-sense	FTLE	No	No	No		(16)
c.3265-3C>T	Splicing	FFE	NA	No	No	66	(17)
c.1092_1099insGGATTTGG p.Val367Gly fs*40		FFE	NA	No	No	66	
c.1625A>C p.Gln542Pro	Missense	FFE	NA	No	No	75	
c.526C>T p.Gln176*	Non-sense	FFE	NA	MI, ID, PA	No	33	
c.1264C>T p.Arg422*	Non-sense	FFE	NA	No	No	66	
c.4033+5A>G	Splicing	FFE	NA	MI	No	50	
c.3461C>T p.Ser1154Phe	Non-sense	FFE	NA	ASD	No	66	
c.492delTCGTT p.Arg165Tyr fs*14		FFE	NA	No	No	50	
c.3241A>C p.Thr1081Pro	Non-sense	FFE	NA	ID	No	50	
c.3696+5G>A	Splicing	FFE	NA	N	Yes		
c.435G>A p.Trp145*	Non-sense	FFE	NA	MI	No		
c.3994C>T p.Arg1332*	Non-sense	FFE	NA	N	Yes	50	
c.727C>T p.Arg243*	Non-sense	FFE	NA	ID	No	50	
c.640C>G p.His214Asp	Missense	FFE	NA	MI	No	50	
c.161A>C p.Gln54Pro	Missense	FFE	NA	ID	No	50	
c.3803G>A p.Arg1268Gln	Missense	FFE	NA	MI	No	50	
c.1909C>T p.Arg637*	Non-sense	ADNFLE	NA	No	No	50	
c.985delA p.Thr329Leu fs*7		FFE	NA	No	No	60	
c.4203+2T>A p.Ala1345GlyfsTer56	Splicing	FFEVF	Yes	No	Yes	100	(2)
c.3311C>T p.S1104L	Missense	FFE	NA	PKD	No	100	(18)
c.4718T>C p.L1573P	Missense	FFEVF	No	ID	No		(19)

FFEVF, familial focal epilepsy with variable foci; FFE, familial focal epilepsy; ADNFLE, autosomal dominant sleep-related hypermotor epilepsy; FTLE, familial temporal lobe epilepsy; RE, Rolandic epilepsy; NA, not available; Y, yes; N, normal; ASD, autism spectrum disorder; ID, intellectual disability; PD, psychiatric disorder; DD, developmental delay; MI, migraine; PA, parasomnias; FCD, focal cortical dysplasia; PKD, paroxysmal kinesigenic dyskinesia. *Stop.

About 52% (11/21) families that carried DEPDC5 mutations were diagnosed with refractory epilepsy. Among them, 81.8% (9/11) families carried non-sense mutations, and 18.2% (2/11) families carried splicing mutations. These studies show that 8.9% (6/67) of families carrying DEPDC5 mutations were diagnosed with autosomal dominant nocturnal frontal lobe epilepsy (ADNFLE). Among them, 75% (3/4) ADNFLE patients carrying DEPDC5 mutations were diagnosed with refractory epilepsy. And 26% (18/67) of families with DEPDC5-related FFE developed FFEVF, and about 50% (2/4) FFEVF patients carrying DEPDC5 mutations were diagnosed with refractory epilepsy.

In these families, about 40% (25/61) of families have other accompanying neurological diseases such as intellectual disability, migraine, autism spectrum disorder (ASD), and psychiatric disorders. About 8% (2/25) of families carrying splicing mutations of DEPDC5 and 64% (16/25) of families carrying non-sense mutations of DEPDC5 have other accompanying neurological diseases.

## Discussion

Recent studies reported that DEPDC5 accounted for 13% of ADNFLE, 2% of Rolandic epilepsy (RE) or BECTS, and a small number of TLE. Patients with DEPDC5-related epilepsy syndromes are more likely to suffer from refractory epilepsy, brain malformation, and mental retardation than others (12, 20). In this study, we reported the clinical characteristics of four focal epilepsy families carrying novel DEPDC5 mutations, including two missense mutations (c.1729G>A, c.3260G>A), one splicing mutations (c.280-1G>A), and one frameshift mutation (c.515_516delinsT). The clinical phenotype includes FFEVF and FFE. In these four families, the onset age of epilepsy ranged from 3 months to 45 years old. Phenotypic intrafamilial variability, like variable lesion onset, is a unique clinical feature of DEPDC5-related epilepsy. Among them, the most common type of epilepsy was frontal epilepsy, accounting for 42.8% of epilepsy. Of our patients, 21.4% had intractable epilepsy. Intellectual disability was identified in 7.1% of our patients.

In our study, some specific mutations contribute to severer clinical manifestation. Compared with other patients, epilepsy patients from family 1 tend to suffer severer epilepsy, and 33% patients from this families have refractory epilepsy, with the onset mainly under 1 year old. All the patients with refractory epilepsy or developmental delay were from this family. Previous studies (Table 2) demonstrated that patients carrying splicing mutations and non-sense mutations of DEPDC5 were susceptible to refractory epilepsy, usually accompanied by other neurological diseases such as intellectual disability, migraine, ASD, and psychiatric disorders, while patients carrying frameshift mutations or missense mutations generally tend to have good prognosis. Non-sense mutation and missense mutation are the most common variants.

The performance of the structural MRI is another character in DEPDC5-related epilepsy. Previous studies (Table 2) demonstrated that only a small number of patients had positive abnormalities in imaging study. However, FCD II was confirmed in some cases with positive MRI findings. Given the role of mTOR pathway in brain development, we speculated that there are tiny lesions in patients with DEPDC5 mutations, but imaging studies were negative. In our study, MRI of all patients in the four families revealed no obvious abnormalities. The possible underestimation of lesions in DEPDC5-related epilepsy is mainly due to the small lesions either in cell morphology or in tissue structure in the brain. The genetic mode of DEPDC5 is autosomal dominant with the penetrance rate ranging from 25 to 100%. The asymptomatic carriers might be explained by incomplete penetrance. The various rates of penetrance still need to be investigated. Genetic anticipation is a character of DEPDC5-related epilepsy, particularly in splicing mutation of DEPDC5. A similar phenomenon has been reported in several studies (14, 15). TSC, MCD, and DEPDC5-related focal epilepsy are classed as mTORopathies. Second-hit theory has already been established well in TSC. Ingrid et al. first put forward the same hypothesis in DEPDC5 mutation-related epilepsy (1). On the basis of germline mutations, there also exists somatic mutation on the other allele or on another gene involved in mTOR in local brain tissue (21–23). The penetrance of DEPDC5 mutation and phenotype of intrafamilial variable foci onset may be determined by the rate of a somatic mutation in local brain tissue.

The phenotype and genotype characteristics of DEPDC5 provide the basis for its treatment. Previous publications reported that 50% of patients have refractory epilepsy; 80% of patients with refractory epilepsy caused by DEPDC5 mutations were seizure-free or had significant improvement after surgical resection (4, 24). Previous studies demonstrated that mTOR inhibitor could achieve long-term seizure reduction and had an overall disease-modifying effect (25), which proved the potential effect of rapamycin in DEPDC5-related epilepsy. Furthermore, some studies pointed out that DEPDC5 agonist selectively affects the activity of mTORC1, without influencing normal metabolism (6). A recent study showed that ketogenic diet (KD) could inhibit mTOR signaling in the brain of rats (26), which suggested that KD might be used to treat intractable mTOR-related epilepsy.

In summary, we reported four novel DEPDC5 mutations identified in four Chinese families with FFE. We found that patients carrying different DEPDC5 mutation have different clinical manifestations. Combined with the reported cases, we found that patients harboring splicing mutations and non-sense mutations tend to suffer from refractory epilepsy and other severer comorbidities. Our study might provide significant information to physician for the treatment and prognosis evaluation of patients with DEPDC5-related epilepsy.

## Data Availability Statement

The datasets presented in this study can be found in online repositories. The names of the repository/repositories and accession number(s) can be found in the article/supplementary material.

## Ethics Statement

The studies involving human participants were reviewed and approved by Xuanwu Hospital Capital Medical University. Written informed consent to participate in this study was provided by the participants' legal guardian/next of kin. Written informed consent was obtained from the individuals and minors' legal guardian for the publication of any potentially identifiable images or data included in this article.

## Author Contributions

XZ and ZH contributed to the conception and drafting of the manuscript. JY, XZ, ZH, JL, ML, XZ, and YW revised the manuscript. JY drafted the manuscript and the final approval version to be published. All authors contributed to the article and approved the submitted version.

## Conflict of Interest

The authors declare that the research was conducted in the absence of any commercial or financial relationships that could be construed as a potential conflict of interest.
